# A Note on the Management of Shoulder Presentations

**Published:** 1885-07-20

**Authors:** E. F. Wells

**Affiliations:** Minster, Ohio


### A NOTE ON THE MANAGEMENT OF SHOULDER PRESENTATIONS.

BY E. F. WELLS, M. D., OF MINSTER, O.

I recently was called to see a woman, aged 44 years, three days in labor with her thirteenth child. She was a well formed woman and had passed through the period of her pregnancy to full term, with no symptoms of any importance. When I arrived I found the cord prolapsed and the left shoulder presenting at the pelvic brim. The os was fully dilated, and the amniotic fluid had long since drained away. The woman had become restless and irritable, was a little feverish, and the vagina and os uteri were swollen, dry, hot, and painful.

The cord was pulseless, but was warm and moist, and the midwife informed me that it had been pulsating only a short time before. I determined, therefore, to replace the cord before proceeding to turn, and to facilitate this I placed the patient in the knee-chest position. The cord was then easily replaced well up in the uterine cavity. In doiug this I was astonished at the facility with which I passed my hand by, and the mobility of the previously firmly wedged-in shoulder; and, pursuing the investigation further, I found that I could readily push up the shoulder, and, by external manipulation, bring down the head, which was done. Maintaining the parts in this position, I directed the patient to rise upright on her knees, and soon had the satisfaction of knowing that the pains were engaging the head in the superior strait. A dose of ergot was administered, the woman placed in a more comfortable position, and the labor was soon terminated in a natural manner, the child being dead. The mother recovered without any trouble whatever.

In this connection, I wish to call attention to the old, but long neglected plan of placing the woman in the "knee-chest" position when about to perform version in cross presentations. In this position the operator has the aid of gravity in relieving the downward pressure upon the pelvic brim, thereby facilitating the passage of the hand into the uterus and of the subsequent manipulations, and, I conceive, tending to prevent the unfortunate accident of rupture of the uterus. I feel confident, also, that cephalic version will be found perfectly feasible in many cases in which podalic version would be the only alternative with the woman in the ordinary position. Another advantage is, that the pains are much less powerful and effective when the uterus is suspended than when it is lying upon the spine and pelvis. I should very much like to see this method of dealing with these difficult cases given a fair and impartial trial by accoucheurs, and the results laid before the profession for future guidance.—*Jour. Am. Med. Ass.*

